# Integrating Circulating Biomarkers in the Immune Checkpoint Inhibitor Treatment in Lung Cancer

**DOI:** 10.3390/cancers12123625

**Published:** 2020-12-03

**Authors:** Boris Duchemann, Jordi Remon, Marie Naigeon, Laura Mezquita, Roberto Ferrara, Lydie Cassard, Jean Mehdi Jouniaux, Lisa Boselli, Jonathan Grivel, Edouard Auclin, Aude Desnoyer, Benjamin Besse, Nathalie Chaput

**Affiliations:** 1 Laboratory of Immunomonitoring in Oncology, Gustave Roussy Cancer Campus, CNRS-UMS 3655 and INSERM-US23, F-94805 Villejuif, France; boris.duchemann@aphp.fr (B.D.); marie.naigeon@gustaveroussy.fr (M.N.); lydie.cassard@gustaveroussy.fr (L.C.); jean.jouniaux@gustaveroussy.fr (J.M.J.); lisa.boselli@gustaveroussy.fr (L.B.); jonathan.grivel@gustaveroussy.fr (J.G.); aude.desnoyer@aphp.fr (A.D.); 2 Faculty of Medicine, University Paris-Saclay, F-94276 Le Kremlin Bicêtre, France; Benjamin.BESSE@gustaveroussy.fr; 3 Hopital Avicenne, Oncologie Médical et Thoracique, Assistance Publique des Hôpitaux de Paris (AP-HP), F-93000 Bobigny, France; 4 Department of Medical Oncology, Centro Integral Oncológico Clara Campal (HM-CIOCC), Hospital HM Delfos, HM Hospitales, 08023 Barcelona, Spain; jordi.remon@delfos.cat; 5 Faculty of Pharmacy, University Paris-Saclay, F-92296 Chatenay-Malabry, France; 6 Cancer Medicine Department, Gustave Roussy Cancer Campus, F-94800 Villejuif, France; Lmezquita@clinic.cat; 7 Thoracic Oncology Unit, Department of Oncology, Fondazione I.R.C.C.S. Istituto Nazionale dei Tumori, 20133 Milan, Italy; roberto.ferrara@istitutotumori.mi.it; 8 Medical and Thoracic Oncology Department, Hôpital Européen Georges Pompidou, APHP, F-75015 Paris, France; edouard.auclin@aphp.fr; 9 Laboratory of Genetic Instability and Oncogenesis, UMR CNRS 8200, Gustave Roussy, Université Paris-Saclay, F-94805 Villejuif, France

**Keywords:** circulating biomarker, immune-checkpoint inhibitor, liquid biopsy, lung cancer, blood biomarker, immunotherapy

## Abstract

**Simple Summary:**

Immune checkpoint inhibitors (ICI) are now a cornerstone of treatment for non-small cell lung cancer (NSCLC). Despite reporting tremendous results for some patients, ICI efficacy remains reserved to a subgroup that is not yet fully characterized. Tissue based assays, such as Programmed cell death protein 1 (PD-L1) expression may enrich the responder population, but this biomarker is not always available or reliable, as responses have been observed in patients with negative PD-L1. Blood markers are hoped to be easier to access and follow, and to give an insight on patient’s immune status and tumor as well. To date, several papers have been looking for circulating biomarkers that are focused on tumor cells or host specific or general immunity in NSCLC treated with ICI. In this article, we review these circulating biomarkers in peculiar circulating immune cell, tumor related cell and soluble systemic marker. We describe the available data and comment on the technical requirements and limits of these promising techniques.

**Abstract:**

Immune checkpoint inhibitors are now a cornerstone of treatment for non-small cell lung cancer (NSCLC). Tissue-based assays, such as Programmed cell death protein 1 (PD-L1) expression or mismatch repair deficiency/microsatellite instability (MMRD/MSI) status, are approved as treatment drivers in various settings, and represent the main field of research in biomarkers for immunotherapy. Nonetheless, responses have been observed in patients with negative PD-L1 or low tumor mutational burden. Some aspects of biomarker use remain poorly understood and sub-optimal, in particular tumoral heterogeneity, time-evolving sampling, and the ability to detect patients who are unlikely to respond. Moreover, tumor biopsies offer little insight into the host’s immune status. Circulating biomarkers offer an alternative non-invasive solution to address these pitfalls. Here, we summarize current knowledge on circulating biomarkers while using liquid biopsies in patients with lung cancer who receive treatment with immune checkpoint inhibitors, in terms of their potential as being predictive of outcome as well as their role in monitoring ongoing treatment. We address host biomarkers, notably circulating immune cells and soluble systemic immune and inflammatory markers, and also review tumor markers, including blood-based tumor mutational burden, circulating tumor cells, and circulating tumor DNA. Technical requirements are discussed along with the current limitations that are associated with these promising biomarkers.

## 1. Introduction

Programmed cell death protein 1 (PD-1) is a transmembrane molecule, which was initially described for its involvement in programmed cell death [1]. PD-1 expression is rapidly induced after signaling via the T cell receptor (TCR) and the level of expression is modulated by cytokines such as IFN-alpha [2,3]. PD-1 regulates both the magnitude and quality of T cell responses, playing a pivotal role in the induction and maintenance of central, as well as peripheral tolerance [4]. This can be seen with antigen presentation of resting dendritic cells, which induces peripheral CD8^+^ T cell tolerance by signaling through PD-1 and CTLA-4 on CD8^+^ T cells [5]. PD-1 acts as a ‘rheostat’ that regulates the threshold, strength, and duration of T cell responses [6]. PD-1 functions as a co-inhibitory receptor during immune response to pathogens and cancer [7], and it counterbalances co-stimulatory receptors on T cells, such as CD28 [8]. PD-1 interacts with two ligands, programmed death-ligand 1 (PD-L1, also called B7-H1) and PD-L2 (also called B7-DC). The pattern of expression of PD-L1 and PD-L2 differs; while PD-L1 is expressed by hematopoietic and non-hematopoietic cells, PD-L2 expression is restricted to hematopoietic cells. In vitro, PD-1 inhibits T cell activation by recruiting SHP2 after binding to its ligands, which leads to the dephosphorylation of crucial tyrosine residues within the CD3 complex and CD28 [7]. The PD(L)1 signaling axis is a cornerstone of immune evasion. The activation of this axis may lead to T cell inhibition, exhaustion, and apoptosis [9].

PD(L)1 checkpoint inhibitors now form part of the backbone of most therapeutic regimens in locally advanced and metastatic NSCLC, either as monotherapy or in combination with chemotherapy, offering significant improvements in the overall survival (OS) when compared with classic strategies. However, in the metastatic stetting, the clinical response is variable despite a favorable toxicity profile and long-term benefit in a proportion of patients. Clinical data showed that blocking PD-1 or PD-L1 in cancer patients could invigorate T lymphocytes, leading to disease control [10]. However, a proportion of treated patients experience a detrimental effect showing a hyper-progressive pattern [11,12]. In the context of the current era of precision medicine, redefining the utility of potential biomarkers is an essential element of the ICI therapeutic strategy; firstly, when selecting patients who will most likely benefit from the different ICI regimens (i.e., ICI monotherapy vs. ICI combination with chemotherapy, or other ICI associations), and secondly, to balance the financial cost that is associated with ICI to ensure the sustainability of our healthcare systems.

To date, PD-L1 expression on tumor cells is the only biomarker that is widely used and approved for selecting patients for PD(L)1 immunotherapy [13,14]. Nevertheless, the focus on tumoral expression of PD-L1 may be too restrictive, given its clonal and spatial heterogeneity, such as is described in early stage NSCLC in the TracerX study [15] and, more recently, in advanced stages [16], that can lead to misinterpretation of PD-L1 status [17]. Moreover, to be effective, antitumoral immunity requires tumor infiltration by immune cells, but, most importantly, an responsive immune system [18]. In many infectious diseases, the host’s immune condition is more widely evaluated by a blood-based test, rather than by biopsy of the infected organ. Baseline biopsies provide an incomplete insight into tumoral burden evolution under treatment. Moreover, biomarkers are needed in order to limit ICI exposure for patients who can develop hyper-progressive disease [11].

On the other hand, circulating biomarkers can act as surrogates for local immune mechanisms, allowing for a more comprehensive evaluation of the host’s immune capacity and longitudinal testing. In this review, we describe the main tissue biomarkers, as well as circulating biomarkers, encompassing immune cells, tumor cells, and inflammatory markers.

## 2. Biomarkers in Tumor Tissue

Although valuable predictive markers have emerged, notably tumoral PD-L1 expression levels, they do not provide a reliable distinction between patients who will respond versus those who will not. In advanced, treatment-naïve NSCLC, PD-L1 expression by immunohistochemistry has been used as the primary biomarker for selecting patients that are more likely to be suitable for receiving ICI, mainly anti-PD(L)1 inhibitors. The survival benefit of ICI as compared with standard platinum-based chemotherapy has been assessed in different settings [19,20,21,22,23]; however, early results were discordant between the different drugs. Studies with nivolumab [19,20] and atezolizumab [23] showed no impact on OS, whereas a significant increase in OS was reported with pembrolizumab as compared with chemotherapy [21,22]. Further analyses showed that the survival benefit that is seen with ICI as monotherapy in the first-line setting is mainly generated in the subgroup of patients with high PD-L1 expression [21,22,23]. On the other hand, in the first-line setting in advanced NSCLC patients, the predictive value of PD-L1 expression diminishes when ICI are combined with chemotherapy, as the survival benefit with the combination over chemotherapy alone is seen, regardless of PD-L1 expression and histologic subtype [24,25,26].

Both microsatellite instability (MSI) and mismatch repair deficiency (MMRD) represent hypermutator phenotypes [27]. MSI and MMRD tumors are highly immunogenic and they have been used as predictive biomarkers for ICI efficacy, proven in clinical trials with anti-PD-1. These trials reported unprecedented overall response rates (ORRs) in different MSI-high/MMRD tumors [28,29], leading to the first therapeutic agnostic approvals by the Food Drug Administration in patients with unresectable or metastatic solid tumors with MSI-high or MMRD, independent of the tumor histology.

Recent data suggest that tumor mutational burden (TMB) could be another predictor of ICI efficacy. The CheckMate 026 trial compared nivolumab with chemotherapy in PD-L1 ≥ 5% in the first-line NSCLC setting. In exploratory subgroup analyses that were based on TMB, higher ORR and longer progression-free survival (PFS) were reported with nivolumab when compared with chemotherapy in tumors with high TMB (ORR: 47% versus 28%; PFS: 9.7 months versus 5.8 months) [19]. Likewise, TMB was used as a prospective biomarker for PFS as one of the two coprimary endpoints in the CheckMate 227 study, which compared the combination of nivolumab and ipilimumab to platinum-based chemotherapy [30]. In the group of patients with a high TMB based on a tissue biopsy (tTMB; defined by ≥10 mutations per megabase [Mut/Mb], accounting for 44% of evaluable patients), a significant benefit in PFS was reported in the combination arm when compared with chemotherapy (7.2 months versus 5.5 months, HR 0.58, 75% CI 0.41–0.81), as well as an improvement in ORR (45.3% versus 26.9%) [30]. The coprimary endpoint of the study (OS in patients with PD-L1 ≥ 1%) was also met, with improved OS with the combination when compared to chemotherapy (17.2 months versus 12.2 months, HR 0.62; 95% CI 0.48–0.78). Interestingly, this benefit occurred, regardless of PD-L1 expression (PD-L1 ≥ 1%; HR 0.79, 95% CI 0.65–0.96; and, in patients with PD-L1 < 1%; HR 0.62, 95% CI 0.49–0.79), and the HRs were similar for both tTMB cut-offs assessed (high tTMB: HR 0.68, 95% CI: 0.51–0.91; low tTMB: HR 0.75, 95% CI: 0.59–0.94), bringing into question the predictive role for tTMB as a biomarker [20]. Similarly, in recent exploratory analysis, tTMB (by whole exome sequencing, defining high tTMB ≥ 175 Mut/exome) was not significantly associated with efficacy of pembrolizumab plus platinum-based chemotherapy or of chemotherapy alone as first-line therapy for metastatic NSCLC, regardless of histology [30], supporting neither the prognostic nor tbe predictive value of tTMB. On the contrary, another exploratory analysis reported an association between higher tTMB levels and improved clinical outcome with pembrolizumab monotherapy in PD-L1-positive NSCLC patients [31], which suggested a synergism with both biomarkers that should be explored in prospective cohorts. However, in the CheckMate 227 trial, the magnitude of the benefit with the combination of nivolumab and ipilimumab did not improve in the subgroup of patients with both high TMB and high PD-L1 [20].

The KEYNOTE-158 study confirmed the clinical activity of pembrolizumab in tumors with a high tTMB (≥10 Mut/Mb) across a variety of previously-treated solid tumors (ORR of 29% vs. 6%) [32]. This led to FDA approval of pembrolizumab in the tumor-agnostic setting with high tTMB; ≥10 Mut/Mb, as determined by an FDA-approved test, after progression on prior treatment.

Today, the level of PD-L1 expression is the main predictive biomarker that is used in routine clinical practice for patients with NSCLC, in the absence of other more optimal biomarkers. Nonetheless, the requirement for evaluating PD-L1 levels in a tumor tissue biopsy, which is not always clinically feasible, limits the routine use of tTMB. Consequently, interest in non-invasive, “easy access” biomarkers, is growing. In addition, blood samples for liquid biopsies may allow for a more accurate and routine assessment of metastatic disease, including acceptable longitudinal sampling for patients.

## 3. Circulating Immune Cells

**Figure 1:**
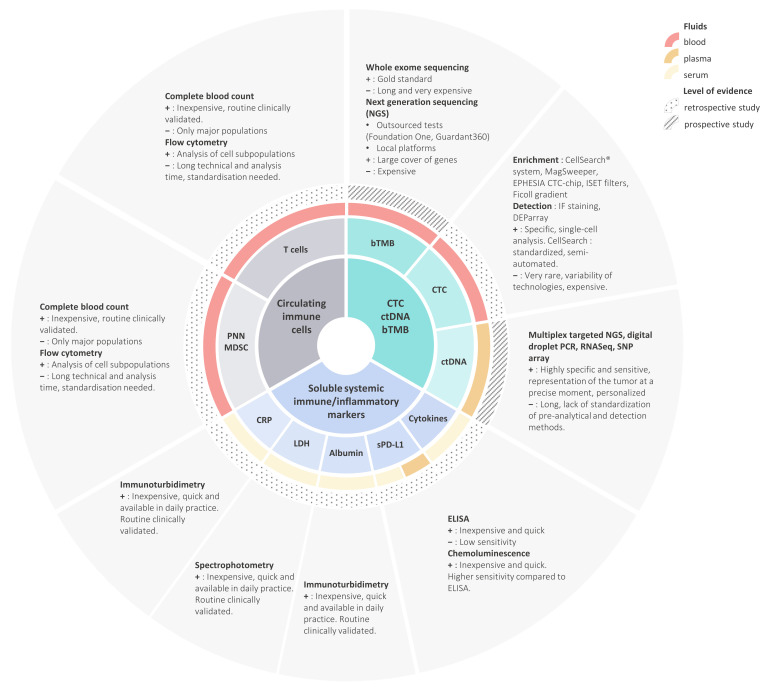
Main biomarkers and technical requirements. Fluids, level of evidence and pros (+) and cons (−) are represented on the third and subsequent layers. ELISA: enzyme-linked immuno assay. NGS: next generation sequencing. PCR: polymerase chain reaction. RNASeq: ribonucleic acid sequencing. SNP: single nucleotide polymorphism.

Blood cells populations, including key immune cell subpopulations, have been historically studied in cancer patients in the context of their role as prognostic circulating markers at diagnosis and baseline. The different immune cells, including T cells, B cell monocytes, and NK cells, are easily quantified in routine management with a complete blood count, which is mandatory at baseline and during therapy for the follow-up of our patients. More precise techniques that allow for the identification of different cell subpopulations by flow cytometry immunophenotyping are also used (Figure 1). Some of these cell subpopulations may be associated with response or toxicity, and they can support the characterization of the mechanism of action, which may be useful for anticipating the optimal combination treatment for future trials. Moreover, blood monitoring during treatment is feasible in real time while using fresh samples, which, for some immune cell populations, is more reliable. Comparative studies using frozen peripheral blood mononuclear cells (PBMC) and fresh whole blood identified that some populations were similarly detected, while others were not consistently determined in frozen PBMC [33]. The T cell populations defined by CD45RA and/or CD62L and by CCR7^+^ and CD45RA^+^ (naive T cells), as well as regulatory T cells (Treg), were underestimated in PBMC, with a variability of 20% to 30%, as were other minor populations (e.g., plasmocytes and dendritic cells) and populations with a heterogeneous expression of markers (e.g., CD14 monocytes, CD16, and CD56). In addition, neutrophils, the major subpopulation of leucocytes, cannot be recovered from frozen PBMCs.

### 3.1. CD3^+^ Lymphocytes (T Cells)

T cells are the most studied cell population in the blood of patients that are treated with ICI, and their predictive value has been addressed in several studies. PD-1^+^ T cells are more frequent in lung cancer patients than in healthy controls [34,35]. PD-1 expression on T cells has also been associated with a worse prognosis in lung cancer. In a study including 42 NSCLC patients, OS and PFS were shorter in patients with a high expression of PD-1^+^ CD4^+^ T cells in blood sample before treatment [34]. In another study, PD-1, PD-L1, and PD-L2 were evaluated in PBMC while using flow cytometry. Among the 70 patients who did not receive ICI, immune checkpoint molecule expression, including PD-1 and PD-L1, was associated with a poor prognosis, in particular PD-L1 expression on CD8^+^ and PD-1 expression on CD4^+^ T cells [35]. The baseline expression of PD-1 on T cell membranes was also associated with poor prognosis in a small patient cohort [34].

In terms of ICI treatment, the early behavior of PD1^+^ populations may be able to predict response. In a study that was published in 2017 by Kamphorst et al., an increase in proliferation of PD-1^+^ CD8^+^ T cells in the blood during the four weeks after treatment initiation was associated with a positive clinical outcome [36]. These proliferating PD-1^+^ CD8^+^ T cells harbored an effector phenotype (HLA-DR^+^, CD38^+^, Bcl-2^low^), expressed co-stimulatory molecules (CD28, CD27, ICOS), and had a high expression of cytotoxic T-lymphocyte-associated protein 4 (CTLA-4). After anti-PD-1 infusion, the early proliferation of PD-1^+^ CD8^+^ T cells was observed in 79% of patients with clinical benefit and only 22% of patients with disease progression. This observation requires further validation, given the small number of NSCLC patients included (*n* = 29).

More recently, a peripheral blood PD-1^+^ CD8^+^ T cell-proliferative response after one week of anti-PD-1 therapy was positively associated with better outcomes in patients with NSCLC [37]. The authors tested Ki-67_D7/D0_ as a predictive biomarker in a cohort of patients with NSCLC and thymic epithelial tumors. In a population of 31 thymic epithelial tumors and 79 NSCLC patients, these T cells proliferated as early as the seventh day after ICI initiation with a diminution after three weeks. The optimal cut-off for Ki-67_D7/D0_ was determined to be 2.8. The probability of clinical benefit was significantly higher in patients with Ki-67_D7/D0_ > 2.8 than in patients with Ki-67_D7/D0_ < 2.8 (*p* < 0.001). The median PFS was 8.7 months (95% CI, 4.3–13.2 months) in patients with Ki-67_D7/D0_ 2.8 and 3.9 months (95% CI, 1.2–6.6 months) in those with Ki-67_D7/D0_ < 2.8 (*p* = 0.027). Of note, a composite score while using tumoral PD-L1 expression did not increase the predictive value [37]. Another study of 31 patients with advanced NSCLC treated with ICI reported that baseline high CD8^+^ PD1^+^ levels in blood and low CD8^+^ PD1^+^ in tissue by immunohistochemistry were associated with better prognosis [38]. These studies highlight the possible role of PD1 expression in circulating T cells, differing from PD(L)1 expression on tissue and it offers a means of early selection of patients that are likely to derive benefit from ICI.

T cell differentiation has been described in cancer patients that were treated with immunotherapy (Figure 1). Patients with metastatic NSCLC have a specific activation profile, with more frequent memory effector T cells and fewer naïve cells [39]. Central to memory effector T cell ratios (TCM/TEM) have been shown to predict response during ICI treatments [39]. In an independent cohort of 22 NSCLC patients, those with high TCM/TEM ratios had longer PFS. In addition, high TCM/TEM ratios was associated with a more pronounced inflammatory signature and high PD-L1 tumoral expression. The same trend was found in another study of 51 patients, with a better response in the cases of more frequent CD4^+^ memory T cell with a low co-expression of PD-1/LAG-3 (Lymphocyte-activation gene 3) population [40]. These studies point towards a need for more memory T cells than activated T cells, which suggests the importance of an available pool of non-effector T cells.

A recent study proposed that clonal expansion of intratumoral T cells can predict therapeutic outcome for immune checkpoint blockade [41]. However, little is known regarding the significance of their peripheral counterparts. In metastatic NSCLC, some TCR are found in both T cells of the tumor and the blood. TCR can be expanded, oligoclonal, in tumors with more than 20% of intratumoral T cells sharing the same TCR [42]. In some cases, persistent expression in the blood of this dominant TCR was associated with persistent response after treatment. In another report, the same trend was found after PD-1 blockade in NSCLC with an oligoclonal expansion of preexisting intratumoral T-cell clones in peripheral blood in responders [43]. For patients who developed acquired resistance after an initial response, the frequency of intratumoral clones decreased in blood at the time of resistance. Interestingly, patients with primary resistance had no such evolution of the TCR repertoire. The importance of these data was highlighted following a recent study demonstrating that specific pre-existing intratumor T cells that are exhausted may have limited reinvigoration capacity, and that T cell response to checkpoint blockade that is derived from a distinct repertoire of T cell clones that may have recently invaded the tumor microenvironment [44]. These results reinforce the importance of evaluating peripheral immune blood cells, as these cells may be recruited and, ultimately, infiltrate the tumoral site. Similarly, a pretreatment dormant tumor infiltrating lymphocytes signature characterized by elevated CD3, but low granzyme-B and low Ki-67, was associated with better disease control rate and longer PFS in NSCLC patients that were treated with ICI, likely due to cytolytic activation/proliferation of dormant T-cells by PD1/PD-L1 blockade [45].

Immunosenescence is a phenomenon that is related to chronic antigenic stimulation. T cell senescence reflects a terminal differentiation status of T cells with low proliferative activity, an oligoclonal TCR repertoire, and reduced capacity to recognize antigenic diversity [46]. Senescent and exhausted T-cells shared some characteristics; however, senescent cells conserve cytotoxic potential [47]. CD28, CD57, and KLRG1 have been identified as markers of T-cell immunosenescence. In a recent study, the percentage of CD28^−^, CD57^+^, and KLRG1^+^ cells among CD8^+^ T-cells (senescent immune phenotype, SIP) was assessed by flow cytometry in blood from patients with advanced NSCLC [48]. A SIP cut-off was identified in a discovery cohort (*n* = 37), with 27% of patients being SIP+. In the validation cohort (*n* = 46), SIP^+^ significantly correlated with worse ORR (0% vs. 30%, *p* = 0.04), median PFS (1.8 months, 95% CI 1.3-NR vs. 6.4 months, 95% CI 2–19; *p* = 0.009), and median OS (2.8 months, 95% CI 2.0-NR vs. 20.8 months, 95% CI 6.0-NR; *p* = 0.02). In the ICI-pooled population (*n* = 83), SIP^+^ status did not correlate with any clinical characteristics and it was associated with significantly worse ORR, PFS, and OS. In the chemotherapy treated cohort (*n* = 61), 11% of patients were SIP^+^, and SIP status did not correlate with outcomes.

Taken together, these studies demonstrate a relationship between circulating immune cells and response to immunotherapy. These results suggest the potential of circulating immune T cells in guiding treatment, but they must be confirmed in prospective studies.

### 3.2. Neutrophils

Recent literature indicates that tumors can exert an influence on neutrophils, in some cases early in the differentiation process, in order to create various phenotypic and functional polarization states, which are, in turn, capable of influencing tumor development. In patients with solid tumors, neutrophils are present in both the tumor microenvironment and in the periphery, and they are generally associated with a poor prognosis [49]. Kargl et al. reported that neutrophils dominate the NSCLC immune landscape and are implicated as potential immune suppressive factors [50]. Recently, a number of circulating innate immune markers, including neutrophils, were shown to be related to outcomes in treatment-naïve advanced NSCLC patients [51]. This study suggested that innate biomarkers were entangled; parameters that are associated with an inflammatory process, such as elevated neutrophils, were related to reduced levels of *NCR3* transcripts and poor NK cell functioning. Thus, neutrophils could subvert antitumor immune effectors and, consequently, might represent an escape mechanism that is linked to poor outcome in patients and resistance to ICI. Neutrophils may modulate treatment efficacy and could also be used as markers of progression or response. The current understanding of the impact of neutrophils on cancer, as well as their mechanisms of action promoting cancer progression and resistance to treatment, is improving. Peripheral blood neutrophil counts are increased in cancer patients, mainly in advanced disease [49]. Tumors produce granulocyte colony-stimulating factor (G-CSF), which skews the neutrophil retention/release balance in the bone marrow, leading to increased neutrophils in the blood [52]. G-CSF downregulates chemokine receptor type 4 (CXCR4) expression in human myeloid lineage cells, which reduces their response to the bone marrow retention signal stromal cell-derived factor 1 (SDF-1) [53].

Biomarkers, such as the neutrophil to lymphocyte ratio (NLR; neutrophils/lymphocytes) and derived neutrophil to lymphocyte ratio (dNLR: [neutrophils]/[leucocytes-neutrophils]), have been investigated to measure inflammatory status in various cancers [49]. A meta-analysis of 100 studies by Templeton et al., including over 40,000 patients, showed that NLR > 4 was associated with worse OS, cancer-specific OS, PFS, and disease-free survival in all types and stage of cancers [54]. However, the choice of threshold of these ratios is not homogenous across reports published in literature. The meta-analysis included trials that were completed before 2013 with limited descriptions of the treatment administered, and it is likely that they included few ICI-based regimens. The NLR has also been evaluated in the immunotherapy setting. In a prospective cohort of 175 advanced NSCLC patients receiving nivolumab in routine practice, NLR ≥ 5 was independently associated with shorter OS [55].

More recently, we examined dNLR along with lactate dehydrogenase (LDH) levels in advanced NSCLC patients that were treated with immunotherapy, which were combined as the Lung Immune Prognostic Index (LIPI). We concluded that high baseline dNLR (>3) and LDH (>upper limit of normality [ULN]) was associated with worse outcomes for ICI treatment (*n* = 466), but not with chemotherapy (*n* = 152) [56]. The LIPI was studied in two pooled cohorts from clinical trials, including immunotherapy, one cohort of 1489 patients that were treated with atezolizumab or chemotherapy [57] and the other of 2440 patients that were treated with ICI or chemotherapy [58]. In both studies, a good LIPI score was associated with better OS among patients, irrespective of whether they received ICIs or chemotherapy. However, the limit between the prognostic or predictive value of NLR and LIPI in NSCLC patients treated with ICI remains unclear. There are still unresolved questions regarding the difference in the magnitude of the benefit for patients treated with immunotherapy [59], and the role of the LIPI score should be better defined in prospective studies. Of note, dNLR can be longitudinally followed with a prognostic value of dNLR evolution during treatment in patients treated with ICI [60].

### 3.3. Myeloid-Derived Suppressive Cells

Myeloid-derived suppressive cells (MDSC), which are a group of myeloid cells with immunoregulatory activity (i.e., suppressed antitumor T cell functions), are composed of two phenotypic/morphologic groups of cells, neutrophil-like (granulocytic (g-MDSC) or polymorphonuclear (PMN-MDSC)) and monocyte-like (M-MDSC) [61]. During chronic inflammatory processes, such as malignancy, there is a persistent signal to recruit neutrophils and monocytes from the bone marrow (e.g., GM-CSF, G-CSF, M-CSF). Over time, the stimulation of the bone marrow is such that the recruited cells are increasingly immature and function aberrantly. These cells are MDSC and, as cancers progress, they form a greater proportion of the circulating cells [62,63,64]. In humans, PMN-MDSCs and neutrophils express both the surface markers CD45^+^CD11b^+^CD15^+^ (or CD66b^+^) and CD14^−^ [61]. A recent study demonstrated that PMN-MDSCs express lectin-type oxidized LDL receptor 1 (LOX-1), which may prove to be a useful marker in distinguishing them from neutrophils [65].

The accumulation of MDSCs is linked to a poor prognosis in patients with NSCLC [64]. MDSC are more frequent in patient with lung cancer than in control patients and they have mainly demonstrated an immunosuppressive function [66]. In patients with advanced NSCLC receiving chemotherapy, increased levels of M-MDSCs correlated with worse PFS (three months versus nine months; *p* < 0.01) [67]. Increased percentages of M-MDSCs were associated with worse ORR (*p* = 0.02) [68]. Data on the prognostic value of MDSC during treatment with ICI are rare. Concerning PD-1/PD-L1 inhibitors, a recent study in two prospective cohorts of 34 (test cohort) and 29 (validation cohort) patients evaluated Lox-1^+^ PMN-MDSC and Treg cells [69]. Before nivolumab administration, no significant difference for Lox-1^+^ PMN-MDSC was observed between the responders and progressing patients. Interestingly, after one injection of nivolumab, Lox-1^+^ PMN-MDSC were diminished in responding patients and an inverse correlation was observed between the percentage of Tregs and Lox-1^+^ PMN-MDSC. The authors evaluated the ratio of Tregs to Lox-1+PMN-MDSCs (TRM ratio) and defined a cut-off value of 0.39, which optimized both sensitivity and specificity. Patients with a TRM ≥ 0.39 had significantly longer median PFS (103 days versus 35 days; *p* = 0.0079) than those with a TRM < 0.39. The authors concluded that PMN-MDSC proliferation and recruitment were markedly higher in non-responders than in responders after anti–PD-1 therapy, which might impair the efficacy of anti–PD-1 therapy.

These studies suggest that inflammatory profiles can be predictive of response. The associated approaches may also provide insight into the mechanism of action and resistance as well as contribute to deciphering the heterogeneity of the setting.

## 4. Soluble Systemic Immune/Inflammatory Markers

The inflammation process has been proposed as a mechanism of immune-resistance in patients with cancer. Numerous routine blood parameters, which were validated for clinical use and inexpensive, have been investigated as potential inflammatory biomarkers in patients with cancer (Figure 1).

### 4.1. LDH, CRP, Albumin and Other Inflammatory Proteins

Unlike normal cells, cancer cells preferentially metabolize glucose by glycolysis in order to generate sufficient energy for rapid proliferation, even in the presence of adequate oxygen. During this process, an upregulation of most of the enzymes involved in the glycolytic pathway, including lactate dehydrogenase (LDH), is observed. In a recent meta-analysis, high LDH was associated with an adverse prognosis in many solid tumors [70], including thoracic malignancies [71] that were treated with chemotherapy, radiotherapy, or surgery without ICI. In a meta-analysis of advanced NSCLC patients that were treated with ICI, high pretreatment LDH levels (defined as LDH >ULN) were significantly correlated with shorter PFS and OS (HR = 1.62, 95% CI 1.26–2.08; *p* < 0.001; HR = 2.38, 95% CI 1.37–4.12, *p* = 0.002, respectively) [72]. This potential prognostic and predictive value of LDH for clinical decision-making warrants further investigation.

Initial systemic inflammation and nutritional status biomarkers are probably less specific, but they may reflect the general condition of the patient and may be important for predicting response to ICI. In retrospective studies, the C-reactive protein (CRP) has been associated with poorer prognosis in NSCLC and other cancers [73,74]. Moreover, in the non-randomized prospective B-F1RST trial evaluating atezolizumab monotherapy in advanced NSCLC, a decrease in serum CRP over six weeks predicted PFS and OS benefits [75]. Regarding nutritional status, low albumin was associated with a poor response to ICI in a retrospective study [76].

Cytokines are another soluble protein of potential interest. In small-cell lung cancer, increased baseline IL-2 levels predicted the sensitivity to ipilimumab, while high IL-6 and TNF-alpha predicted resistance [77]. The dynamic evolution of IL-8 during ICI treatment in NSCLC patients has been reported with greater benefit among patients with early decreases of IL-8 levels [78], while increased tumor necrosis factor or interferon-gamma during ICI treatment correlated with improved response and longer OS [79,80].

### 4.2. Soluble PD-L1

Many surface molecules assume two forms of expression: membrane-bound protein and a soluble form generated after proteolytic cleavage [81]. This is the case for immune checkpoints such as PD-1, PD-L1 and CTLA-4. High serum concentrations of soluble PD-1 (sPD-1), PD-L1 (sPD-L1), and CTLA-4 (sCTLA-4) have been associated with autoimmune diseases [82], and their role in cancer is under investigation. These soluble forms increase the complexity and diversity of the composition and function of the PD-1/PD-L1 signaling pathway [83]. Several cellular types can produce sPD-L1. PD-L1^+^ cell line supernatant contains more sPD-L1 than that seen with PD-L1^−^ [84]. Of note, myeloid cells production of sPD-L1 seems to be more potent than those of the T cells [85]. An absence of correlation between tumor PD-L1 expression and sPD-L1 levels in other cancer types suggests the importance of the microenvironment, including non-malignant cells, in sPD-L1 production [80,86].

Recent meta-analyses have reported sPD-L1 levels in blood of various malignancies and high levels of sPD-L1 significantly predicts poor prognosis in patients with solid tumors [87,88]. In advanced NSCLC, plasma sPDL1 levels are not correlated with histological subtypes or smoking history [89], but the expression of sPD-L1 or sPD-L2 was significantly higher when compared with healthy controls, and high sPD-L1 expression significantly correlated with poor prognosis [35,89,90].

Among NSCLC patients that were treated with anti-PD1 (nivolumab), clinical benefit (complete response, partial response, plus stable disease), and longer OS were more frequently achieved in NSCLC patients with low plasma sPD-L1 levels than in those with high levels (>3.357 ng/mL) [91]. The dynamic evolution of sPD-L1 correlates with treatment efficacy. In a cohort of 43 advanced NSCLC patients that were treated with nivolumab, high sPD-L1 levels at two months after treatment initiation, as well as increased sPD-L1 concentrations during treatment were associated with poor response and an absence of clinical benefit [80]. An optimized cut-off of 33.97 pg/mL sPD-L1 was associated with a sensitivity of 94%, a specificity of 56%, a positive predictive value of 70%, and a negative predictive value of 90% to predict response at the first tumor evaluation. Although the levels of sPD-L1 that were assessed by ELISA in serum and/or plasma could serve as biomarkers, technological issues over sensitivity and reproducibility may limit their clinical application. An automated measurement system based on a chemiluminescent enzyme immunoassay (HISCL system) has been developed in order to overcome these limitations [92], but the optimal technique and cut-off are still to be determined.

Tumor-derived exosomes contain substantial amounts of biologically active proteins, lipids, and nucleic acids acquired from their parental cells, which they can transport to other cells. Exosomes that are derived from lung cancer cells express PD-L1 and play a role in immune escape by reducing T-cell activity and promoting tumor growth. The amount of PD-L1 on these vesicles can impair immune functions by reducing cytokine production and inducing apoptosis in CD8^+^ T cells, which indicates that tumor-derived exosomes expressing PD-L1 may be an important mediator of tumor immune escape [93]. In a mouse xenograft model, exosomal PD-L1 can promote a tumor immune escape mechanism that was abolished by PD1/PD-L1 blockers. Baseline exosomal PD-L1 may also have a negative prognostic value [94]. These observations may provide a rationale for the application of exosomal PD-L1 as a predictor for anti-PD-1 therapy [94].

## 5. Blood Tumor Mutational Burden, Circulating Tumor Cells, and Circulating Tumor DNA

### 5.1. Blood-Based Tumor Mutational Burden

It remains unclear whether TMB estimated while using circulating tumor DNA (ctDNA) in blood (bTMB) is associated with clinical outcomes of ICI. Table 1 describes studies evaluating the predictive value of bTMB in advanced NSCLC. bTMB that is determined by NGS has been reported to be correlated with tTMB using whole exome sequencing [95,96]. In the retrospective exploratory analysis of the POPLAR and OAK clinical studies, it was demonstrated that high bTMB (≥16 Mut/Mb), measured while using the Foundation Medicine assay, was associated with better outcome (PFS and OS in POPLAR and OS in OAK trial), which suggested that bTMB may predict the benefit of atezolizumab as second-line therapy in NSCLC [96]. The MYSTIC trial assessing the efficacy of the durvalumab and tremelimumab or durvalumab alone against first-line platinum-based chemotherapy in a PD-L1-expressing population (PDL-1 ≥ 25%) did not achieve the three primary endpoints, PFS and OS for the immunotherapy combination compared to chemotherapy, and OS for durvalumab compared to chemotherapy. However a retrospective exploratory analysis revealed a survival benefit for durvalumab and the durvalumab plus tremelimumab combination in the subgroup of patients with high bTMB (≥16 Mut/Mb [97] or ≥20 Mut/Mb), with the greatest magnitude of benefit being observed in patients that were treated with the immunotherapy combination [98]. In addition, bTMB was used as a prospective biomarker for the efficacy of first-line atezolizumab monotherapy in advanced NSCLC patients in the non-randomized B-F1RST trial. According to bTMB, ≥16 vs.  <16, median PFS was 5.0 months versus 3.5 months (HR 0.80, 90% CI 0.54–1.18) and OS was 23.9 months versus 13.4 months (HR 0.66, 90% CI 0.40–1.10). These results suggest a clinical benefit, albeit not statistically significant, of atezolizumab in tumors with high bTMB [75]. A confirmatory phase 3 study (BFAST, NCT03178552) assessing the predictive role of bTMB is currently recruiting patients. The phase 3 NEPTUNE trial compared the combination of durvalumab and tremelimumab versus chemotherapy as a first-line treatment. The trial was performed in an all-comers population, and the primary analysis population was patients with a high bTMB (≥20 Mut/Mb). However, in the primary analysis population, the combination did not meet the primary endpoint of improving OS when compared to platinum-based chemotherapy [99].

The dynamic evaluation of TMB is another potential area for which biomarkers can be exploited. In melanoma patients, during treatment with nivolumab, the mutational load decreased in responding patients with a concomitant increase in the T cell oligoclonal profile [100]. Currently, the predictive biomarker role of tTMB or bTMB remains a challenge in the absence of prospective proof of the predictive value for survival, a consensual cut-off defining high TMB, the value of tissue versus blood, and the optimal techniques and platforms for calculating tTMB and bTMB (Figure 1).

### 5.2. Circulating Tumor Cells and Circulating Tumor DNA

The need for non-invasive biomarkers is increasingly important, for both treatment decisions and the dynamic evaluation of treatment efficacy with ICI. ctDNA can be used as a real-time marker of response due to its short half-life [101]. An analysis of circulating tumor cells (CTC) and ctDNA in NSCLC are non-invasive tools for assessing PD-L1 status [102,103] and the dynamic biomarkers of ICI efficacy [43,103,104]. In regarding circulating tumor DNA, several information may be available as the type of mutation (loss-of-function, nonsynonymous Single nucleotides variant, gene fusions…), and somatic or germinal origin by using the variant allele frequency (VAF) [105].

In one study in cancer patients, which included NSCLC patients, with undetectable ctDNA levels after two months of ICI, a marked and lasting response to therapy was presented [106]. Another study reported a similar outcome for patients experiencing an early decrease in ctDNA burden after one month of ICI [107]. Similarly, a >20% increase in ctDNA over the first six weeks of nivolumab therapy predicted worse survival in pretreated patients with advanced NSCLC [108]. These results were confirmed in a large retrospective study of pan-cancer patients that were treated with ICI [109]. ctDNA at baseline and under ICI was investigated in 978 ctDNA samples (including 333 lung cancer samples) at baseline and 171 on-treatment. The pretreatment variant allelic frequency was inversely correlated with OS, and as ctDNA increased during treatment.

As liquid biopsies may capture PD-L1 expression heterogeneity, spatial and temporal tumor heterogeneity [110] have been proposed as potential explanations for a lack of PD-L1 expression concordance between tissue and CTC, which suggested that CTC may provide a more accurate predictive capability than tissue to capture false-negative PD-L1 expression in tissue. However, some limitations are associated with the use of CTC. Technology for CTC isolation is not broadly available and the success of obtaining samples from NSCLC patients ranges from 45% [103] to 93% [102] of samples, with, furthermore, a variable range of PD-L1-positive CTC. Finally, to date, no trials have clearly correlated PD-L1 expression on CTC prior to ICI therapy as a predictive biomarker of efficacy [102,103,111,112,113]. Indeed, two of these studies showed that pre-treatment of PD-L1-positive CTC was associated with a bad prognosis in patients that were treated with anti-PD(L)1 [102,113], with another study reporting an increase in PD-L1-positive CTC upon disease progression, which suggested that it may predict resistance to ICI [112]. Finally, cost and turnaround time may also limit the clinical applicability of CTC in daily clinical practice.

## 6. Composite Score

One of the strengths of blood-based biomarkers is the feasibility of combining evaluations of multiple biomarkers without a material limitation. A number of studies have used a multimodality approach combining several biomarkers.

In a study of 109 NSCLC patients, sPD-L1 and CD8^+^ PD1^+^ were evaluated as biomarkers [114]. High sPD-L1 and low CD8^+^ PD-1^+^ and NK counts were associated with a negative impact on PFS (*p*  <  0.001), OS (*p*  <  0.01) and response (*p*  <  0.05) with ICI. These biomarkers were associated in the immune effector score. In patients with both low sPD-L1 and high CD8^+^ PD1^+^, the median PFS and OS were not reached in contrast to patients with high sPD-L1 or low CD8^+^ PD1^+^ (PFS, HR 5.72, 95% CI 2.17–15.04, *p* < 0.001; OS, HR 5.07, 95% CI 1.71–14.97, *p* < 0.001). The ROC curve for ICI response displayed an AUC value of 0.80 (95%CI 0.66–0.92, *p* = 0.001). More recently, in a cohort of 99 NSCLC patients that were treated with ICI, low CD8^+^, and high bTMB at baseline, and early decrease in ctDNA were associated with durable clinical benefit after one infusion. These parameters were also associated with a score (DIREcf-On). Patients with higher DIREct-On scores had longer PFS than those with lower scores (*p* < 0.0001, HR = 8.93, AUC = 0.93) [115]. These studies show the feasibility of combining biomarkers in order to assess both tumoral and host characteristics at the same time and increase the predictive power of these tests.

## 7. Discussion

Circulating biomarkers encompass different sample types, several techniques, and they focus on different aspects of the immune system at both the tumoral and host level (Figure 1). In summarizing the main results for each biomarker subtype, the presence of circulating PD1^+^ CD4^+^ immune cells or a senescent population at baseline appears to be associated with a bad prognosis, high baseline CD8^+^ PD1^+^ levels or an increase during treatment may be a good early biomarker of response, decreases in ctDNA and bTMB during treatment appear to be associated with response, while high baseline sPDL1 or sPDL1 elevation during treatment also seem to be indicative of worse prognosis. Blood-based tests may be useful in cases of tumoral heterogeneity, such as PD-L1 assessment, as well as providing complementary information on the pool of circulating lymphocytes that can be recruited [44].

This wide range of parameters exploring different aspect of the immune response can be combined in a score in order to improve the strength of the prognostic value [38,115].

Nevertheless, some pitfalls remain regarding these circulating biomarkers that have delayed their use in the clinical settings. Most studies are retrospective or with small population. Large case series with prospective and standardized methodological approaches are mandatory in obtaining reproducible results. To date, no randomized trials evaluating treatment decisions that are based on circulating biomarker have been launched. Most data come from retrospective studies (Table 1); however, prospective ongoing trials should provide some answers (F-1RST NCT02848651, B-FAST (NCT03178552), and the results are eagerly anticipated.

## 8. Conclusions

Circulating biomarkers are promising in the field of antitumor immunotherapy, even though they are not yet systematically leveraged in clinical practice. Some systemic biomarkers are readily available, such as LDH or dNLR; however, specific techniques are required for other biomarkers, such as TMB, lymphocyte, and other immune cell subtypes analysis. These techniques and reporting methods (notably the choice of threshold) must be standardized. These circulating biomarkers can reflect tumoral characteristics and additionally provide information on the host’s immune status. Systemic evaluation can also offer a more comprehensive evaluation of tumoral polymorphism. Moreover, all of these examinations can be longitudinally performed without exhausting tumoral samples and providing early information. An evaluation of these non-invasive biomarkers in prospective clinical trials is essential for their implementation in guiding routine clinical practice.

## Figures and Tables

**Table 1:** Main studies using circulating biomarkers in advanced non-small cell lung cancer (NSCLC) to monitor or guide immune checkpoint inhibitors (ICI) treatment.

Reference	*n*=	Design	Validation Cohort	Therapy	Result/Threshold
**bTMB by NGS**					
[95] Wang 2019	48 + 50	R	Y	ICI, non-specified	bTMB is positively associated with ICI efficacy threshold 6 Mut/mb
[96] Gandara 2028	OAK = 273 POPLAR = 583	R	Y	Atezolizumab	bTMB is positively associated with PFS under atezolizumab therapy threshold 16 Mut/mb
[98] Peters 2019	*n* = 809 Mystic	R	N	Durvalumab+ tremelimumab	bTMB is positively associated survival benefit under combo therapy threshold 20 Mut/mb
[115] Nabet 2020	*n* = 99	R	Y	ICI	High blood based TMB, CtDNA decreased after one infusion, low CD8 are associated with good DCB
**ctDNA by NGS**					
[104] Goldberg 2018	28 analysed on 49 tested	P	No	ICI, not further specified, CTx	ctDNA diminution associated with prolonged survival threshold ctDNA response as a >50% decrease
[106] Cabel 2017	10/15	P	No	Nivolumab	8w decreased of ctDNA
[107] Guibert 2019	67/86	R	No	ICI	Increase vs. decrease in allele fraction
[109] Zhan 2020	333	R	No	ICI	Pretreatment Variant Allelic Frequency was inverse correlated with OS ctDNA increased during treatment was correlated with poor OS
[115] Nabet 2020	*n* = 99	R	Y	ICI	High blood based TMB, CtDNA decreased after one infusion, low CD8 are associated with good DCB

Ref = Reference, *n* = number of patient, R/P retrospective/prospective design, ICI = immune checkpoint, cfDNA = cell free DNA, ctDNA = circulating tumor DNA, NGS = next generation sequencing, bTMB = blood tumor mutational burden, PFS = progression free survival, PBMCs = peripheral blood mononuclear cells, CTx = chemotherapy.
